# Cognitively Inspired Federated Learning Framework for Interpretable and Privacy-Secured EEG Biomarker Prediction of Depression Relapse

**DOI:** 10.3390/bioengineering12101032

**Published:** 2025-09-26

**Authors:** Sana Yasin, Umar Draz, Tariq Ali, Mohammad Hijji, Muhammad Ayaz, El-Hadi M. Aggoune, Isha Yasin

**Affiliations:** 1 Department of Computing, Univeristy of Okara, Okara 56300, Punjab, Pakistan; sana.yasin@uo.edu.pk (S.Y.); ishayasin42@gmail.com (I.Y.); 2 Department of Computer Sceince, University of Sahiwal, Sahiwal 57000, Punjab, Pakistan; 3 Artificial Intelligence and Sensing Technologies (AIST) Research Center, University of Tabuk, Tabuk 71491, Saudi Arabia; 4 Faculty of Computers and Information Technology, University of Tabuk, Tabuk 71491, Saudi Arabia

**Keywords:** personalized federated learning (PFL), EEG-based depression relapse prediction, explainable artificial intelligence (XAI), adaptive wavelet entropy filtering (AWEF), layer-wise relevance propagation (LRP)

## Abstract

Depression relapse is a common issue during long-term care. We introduce a privacy-aware explainable personalized federated learning (PFL) framework that incorporates layer-wise relevance propagation and Shapley value analysis to provide patient-specific interpretable predictions from EEG. The study is conducted with the publicly available Healthy Brain Network (HBN) dataset, with analysis conducted for n = 100 subjects with resting-state 128-channel EEG with accompanying psychometric scores, and subject-wise 10-fold cross-validation is used to assess the performance of the model. Multi-channel EEG features and standardized symptom scales are jointly modeled to both increase the clinical context of the model and avoid leakage issues. This results in overall accuracy, precision, recall, and F1-score values of 92%, 91%, 93%, and 90.5%, respectively. The attribution maps from the model suggest region-anchored spectral patterns that are associated with relapse risk, providing clinical interpretability, and the federated setup of the model allows for a privacy-aware training setup that is more easily adaptable to multi-site deployment. Together, these results suggest a scalable and clinically feasible approach to trustworthy relapse monitoring with earlier intervention.

## 1. Introduction

Depression is the main cause of disability around the world, affecting 280 million people annually. Standard treatments for depression still leave too many patients without hope, with up to 60% of people having a second episode after their first, and 80% of people who have had two or more episodes go on to relapse [1]. It has been estimated that about 50% of patients with one episode of major depressive disorder will have a second one and that the frequency of recurrence increases much more than in previous episodes [2]. According to studies [3,4], the probability of a new relapse after the first is 70%, and, after the third episode, the probability increases to 90%. Relapse in depression has different causes, including incomplete recovery, residual symptoms, stress, and biological vulnerabilities. Prediction of relapse at an early stage enables the alleviation of the effects of repetitive episodes on patient quality of life. Several studies have used an electroencephalogram (EEG) for the detection of depression because it is an inexpensive, non-invasive, and objective technique to identify patterns of brain activity related to depression [5,6]. It is beneficial for mild and moderate to severe major depressive disorder. The ability of this modality to be used in real time allows therapists to track current neural signals and predict early signs of relapse therapy.

Depression is a prevalent and serious mental illness that causes a negative impact on mood, cognition, and daily functioning. It is also a neurologically manifested disorder that is characterized by altered activity in neurochemical, neurophysiological, and electrophysiological domains. As such, biomedical signal analysis has become an active field of research dedicated to understanding depression. In the clinical study of depression, two related tasks are commonly of interest: the detection and prediction of depressive relapse. In this context, detection is typically the problem of determining whether relapse-related neural or behavioral signal patterns are present at the time of data acquisition, and prediction is the problem of estimating the probability of a future relapse episode. These two tasks may also be distinguished when considering EEG data specifically, such that the real-time detection of abnormal brainwave activity (detection) may also be used to supplement active relapse risk forecasting (prediction). In this study, we adopt these specific definitions to make the distinction between detection and prediction tasks, for the purpose of maintaining clarity and consistency, while introducing methods for EEG-based biomarker detection and relapse prediction [7,8]

Major depressive disorder is a phenotype of interacting neurochemical and circuit-level changes that modulate the cortical postsynaptic potentials (PSPs) composing the scalp EEG. These include, in addition to monoaminergic (serotonin, noradrenaline, dopamine) alterations, converging evidence for excitatory–inhibitory imbalance (increased glutamatergic tone and decreased GABAergic inhibition), HPA dysregulation and its downstream cortisol effects on prefrontal–limbic circuits, neuroinflammatory signaling and the downregulation of neurotrophic support, etc. These mechanisms reconfigure the underlying microcircuit dynamics, which are manifested in EEG as, e.g., frontal alpha asymmetry, increases in frontal beta power, aberrant midline/theta activity, sleep EEG anomalies, and dampened event-related potentials (e.g., reduced P300), with directionality and effect sizes modulated by region, subtype, comorbidity, and other factors. Clinically, in addition to mood, depression has consequences for attention/executive control/memory, and, in late-life depression, these functional deficits, along with a vascular/inflammatory burden, are considered causes of increased risk for dementia. Structural and functional MRI have been applied in major depressive disorder to a much greater degree than electrophysiological signatures, which are more directly linked to synaptic dynamics. The latter remain comparatively underutilized to predict the risk of relapse; motivated by this, we utilize resting-state EEG to learn interpretable, physiologically anchored biomarkers of relapse risk using a privacy-preserving, federated approach [9].

Hemispheric lateralization is region- and task-selective, rather than global. Handedness indexes motor cortex asymmetry but does not define overall ‘left’ or ‘right brain’ dominance. Cortical systems can be asymmetric in different directions; for instance, language networks are left-lateralized, visuospatial attention is often right-lateralized, and there are mixed left–right patterns of asymmetry in prefrontal regions associated with affective control. In line with these views, we interpret EEG asymmetry features only within the cortical regions from which they originate (e.g., frontal alpha asymmetry as a putative marker of approach–withdrawal balance) and we do not use handedness to infer overall brain dominance. Handedness is included in the statistical models as a covariate and we report a sensitivity note verifying that inferences drawn based on region-specific EEG features do not depend on handedness.

Recent advances in the prediction of depression relapse have been facilitated by a variety of techniques and are closely related to machine learning and neuroimaging. A deep learning-based approach was used in [7] to solve two problems related to depressive disorders, namely the recognition of depression and prediction of relapse. The model utilizes a ’model of normality’ concept, which implies measuring the proximity of audiovisual patterns during nondepressed episodes to those of listless patients, and it has been tested in a notable DAIC-Woz database. It is considered a flexible and scalable solution for tracking depression and predicting its relapse. In a recent article [8], a new approach to diagnosing depression and predicting a relapse of mental illness is discussed based on videos obtained during clinical interviews. The method is used to determine the degree of similarity in the audiovisual patterns of the subject to those of an already depressed patient, which is typical for video material coming from a subject in clinical practice. As a result, with accuracy of more than 80% in the DAIC-Woz datasets, this represents an effort to predict depression without using user input.

Prior EEG work in depression reports frontal alpha asymmetry (greater right than left alpha), elevated frontal beta power, and altered midline/theta activity, although the exact direction and effect sizes vary by cortical region, clinical subtype, and comorbidity [10]. Under acute stress, frontal-lobe activity—especially within prefrontal–cingulate circuits—can increase, but responses are heterogeneous across subregions and cell populations; accordingly, we interpret stress effects using region-specific EEG features rather than assuming uniform neuronal activation.

The key limitation in existing EEG-based relapse detection methods is the lack of explainability [11]. In clinical settings, knowing why a model makes any specific prediction is important to gain trust from clinicians and to ensure that system recommendations are actionable. New posture data-oriented explainable artificial intelligence (XAI) technologies include layer-wise relevance propagation (LRP) and Shapley values, from which EEG features or brainwave patterns that contribute most to predicting depression rebound can be visually deduced. When XAI becomes part of the relapse detection framework, its clinical adoption can improve the clarity of doctors regarding the underlying neural mechanisms. In addition, there is a growing trend in applying XAI in medical diagnosis to realize better patient outcomes and to make AI models accountable and interpretable. A piece of recent work highlights the importance of bringing XAI to mental health applications, particularly for sensitive tasks such as relapse prediction, where transparent decision making can help clinicians to take appropriate and timely action.

In this study, we present a novel architecture combining personalized federated learning (PFL) and explainable AI (XAI) for the detection of depression relapse based on EEG data. Under the proposed framework, each patient’s model is customized to his or her unique brainwave activity, and the patient’s information is kept personal. Through meta networks and local fine-tuning, the global model picks up the general tendency within multipatient data, whereas each local model tailors its prediction to the specifics of individual data. In addition, XAI techniques provide clarity and understanding, with the result that medical professionals can immediately begin working with these new insights. This twin emphasis on personalization and explainability marks a significant advance in EEG-based depression relapse detection above and beyond the latest findings in personalized medicine and understandable models. Furthermore, incorporating FL into actual clinical practice shows great potential to push patient-centered care another step forward. It ensures that our models always adapts to the evolving needs of patients, and privacy is still maintained at the highest level.

### Contributions

There are three novel contributions to the research on explainable AI for the personalized detection of depression relapse using electroencephalogram (EEG) data.

1. **Electroencephalogram-Based Depression Relapse Prediction:** EEG has been thoroughly investigated for the detection and diagnosis of depression, but no study has been conducted for depression relapse. Few studies have attempted to develop models capable of predicting the time to relapse in depression using audiovisual characteristics [7]. To our knowledge, the prediction of depression relapse using EEG does not yet exist in the literature. Only one article exists in the literature, which was written by one of our authors [5]. The construction of EEG-based relapse prediction models will provide a great opportunity for early intervention and custom treatment plans.
2. **Integration of Personalized Federated Learning (PFL):** The research describes the approach to integrate PFL, where the EEG data of each patient can be kept private. In this way, the data cannot be misused, and precalculated properties can be sent to clients without sharing confidential raw data. This allows for customization to the individual patient, called meta-learning and fine-tuning locally, but it benefits from the global model’s general knowledge, which improves both the effectiveness and privacy in relapse detection for depression.
3. **Layer-Wise Relevance Propagation (LRP) and Shapley Values for Explainability:** Crucial to this is the use of explainable AI features such as layer-wise relevance propagation (LRP), contributing to the interpretability of data. Millions of EEG segments in turn contribute to model predictions, and Shapley values clarify electroencephalography (EEG) results, enabling an understanding of how certain EEG features affect our models’ decisions. Due to this interpretability, executives are more likely to build trust in AI-fueled decisions, which is necessary in healthcare to ensure that clinicians are able to interpret and act on predictions.
4. **Adaptive Wavelet Entropy Filtering (AWEF):** In this study, we propose a new preprocessing method called adaptive wavelet entropy filtering (AWEF) to improve the prediction of depression relapse based on EEG. Combined with a privacy-preserving federated learning framework, it reduces noise from EEG signals while maintaining the essential characteristics of individual signals, thus improving model interpretation and performance. This strategy guarantees accurate and consistent predictions that address the challenges of noise and variability as the use of decentralized mental health diagnostics increases.
5. **Better Predictive Performance for Depression Relapse Detection:** The proposed model shows improved performance on EEG datasets, with precision of up to 92% and an F1-score of 90.5%. These evaluation results indicate that our proposed model could have a superior ability to highly accurately predict depression relapses, as well as to provide more trustworthy, customized, and explainable predictions than existing centralized models. This helps in early and accurate interventions within mental health care.

To our knowledge, there are no EEG-based depression relapse prediction approaches in the literature. Therefore, these contributions underscore the distinctive methodological principles by which privacy-aware personalization and explainability can be targeted for mental health AI solutions.

The paper consists of the following sections. In the Introduction, the relevance of depression relapse identification and the novel PFL-XAI model are discussed. Related Work identifies gaps in existing research with respect to personalization, privacy, and explainability. The Methodology section describes the proposed model, including the personalized federated learning and XAI approaches, while the Results and Discussion section analyzes the model’s results in terms of its precision, F1-score, and explainability. Finally, the Conclusions summarize the main findings and consider the practical implications of the developed method for clinical settings.

## 2. Related Work

In the past few years, several machine learning and deep learning models have been developed to detect depression using EEG data. Several other modalities (MRI, fMRI, MEG, MRS) have also been used to assess neurochemical and structural changes related to relapse, but they are often expensive, less portable, and do not provide the high temporal resolution needed to capture more rapid electrophysiological changes. In contrast, EEG is low-cost, non-invasive, and widely accessible and offers a millisecond-level temporal resolution. This makes EEG a particularly pragmatic modality for use in clinical relapse detection and prediction. In line with the reviewed literature (Table 1), current AI methods for depression relapse analysis can be conceptually classified into three general categories: (i) machine learning approaches that rely on hand-crafted EEG features (e.g., SVM, random forest), (ii) deep learning approaches that utilize hierarchical representation learning (e.g., CNN, LSTM, hybrid models), and (iii) generative AI methods for improved data augmentation and representation learning. This classification offers a structured summary of the field without being overly redundant in narrative.

In [18], an evidence-based mega-analysis, with connectivity abnormalities in youth depression, used the NeuroDatabase site-aggregated neuroimaging data and performed statistical and machine learning analyses on brain images to separate and identify depressed and healthy groups. In recent years, a large number of studies have been conducted to detect depression, primarily using EEG. Some of these techniques include MFCC features with CNNs [19], an LSTM–Attention model [20,21,22], hybrid EEG–NIRS with SVMs [23], and adaptive dynamic convolution [24]. More recent and complex methods include MAST-GCN [25], deep neural networks [26], neurofuzzy methods [27], and LSDD-EEGNet [28]. In addition to EEG, research on other approaches, like mindfulness-based therapy [29,30], maintenance antidepressant treatment [31] , and wearable activity/sleep monitoring [32], has been applied to depression relapse prevention. A systematic review article also underlined artificial intelligence applications for predicting relapse and suicidality in bipolar disorder [33].

In this research, we sought to overcome some of the limitations of these works by using a novel neural architecture and datasets that have not been previously used for post hoc analyses with appropriate performance evaluation metrics. In addition, we focused on women and children as the population for the current study and also used the DSM-5 criteria. A 2.6-billion-word corpus for extended text generation analysis was also a part of our study. Table 2 presents a comparison of studies (2019–2024) on EEG-based depression detection based on personalization, privacy, explainability, temporal/spatial pattern recognition [34,35,36], scalability, and the type of model. From the results, it is obvious that CNNs, LSTM, and reinforcement learning perform the best in temporal/spatial detection but with a lack of personalization, privacy, and explainability.

Table 3 shows a demonstration of the challenges faced by the proposed PFL-XAI model in EEG-based depression detection, contrary to previous solutions. It involves various aspects, such as data privacy, explainability, personalization, scalability, heterogeneous EEG data, and real-time application, as discussed. Previous centralized models are at risk of compromising data privacy [11,15,17], while the PFL approach used in the current model keeps data local and is executed in collaboration without compromising quality. This model further integrates XAI, as opposed to existing XAI and black-box models that limit the explainability of XAI features [12,13]. Similarly, the already generalized methods were not tailored to specific patient needs, limiting personalization [10,14,16]. Furthermore, previous solutions came with high computational costs that made it impossible to use them for real-time applications, although they used high-performance techniques and reinforcement learning [15,17,21]. Furthermore, only the current model is designed to address the heterogeneous sources of EEG data involved. Unlike the previous CNN and LSTM models [10,12,16], the current solution presents a complete data source comparison with improved accuracy. Table 3 provides a concise summary of recent work on the topic of EEG and related modalities for the analysis of depression relapse. Interpretability and robustness have been improved by a variety of methods, including spectral analysis, concept-based XAI, and attention-guided saliency maps. Despite some notable exceptions, most works are based on limited sample sizes, with a lack of relapse follow-up and/or clinical validation and generalizability.

## 3. Dataset

In our research, we utilized a public, de-identified dataset from the Healthy Brain Network (HBN). N = 100 individuals who had complete EEG and clinical information for modeling in the current analyses were included (49 females, 51 males; age at EEG acquisition, 13.7 ± 3.2 years, range 8–20). Although having multiple sources of EEG data often implies different acquisition protocols (sampling frequency, number of electrodes, referencing, and preprocessing pipelines), the data used in the current study were acquired through the HBN, where the same standardized protocol was followed to make the data comparable across all participants. Moreover, the suggested preprocessing pipeline (ICA-based artifact correction and adaptive wavelet entropy filtering) was chosen specifically to limit any protocol-specific noise and variations. The architecture of the proposed model was designed to be easily scalable and can be adapted for extension to multi-site/heterogeneous datasets with different acquisition protocols, pointing out the robustness and scalability of the proposed model for real-world clinical use. EEG was collected as part of the HBN protocol, using a 128-channel EGI HydroCel system and sampled at 500 Hz with impedances maintained <50 kΩ. Signals were referenced to Cz during acquisition and re-referenced to the average offline. Recordings were obtained in a quiet, dimly lit room with participants seated. The protocol included resting-state blocks with eyes open with central fixation and eyes closed, approximately 5 min each; our analyses use these resting-state segments only. Preprocessing included band-pass filtering 0.5–40 Hz bad-channel detection/interpolation, ICA-based artifact attenuation, epoching to task/rest events, baseline correction, and objective trial rejection; only trials meeting quality approval were modeled. Depression diagnoses were taken verbatim from clinician-administered KSADS-C OMP (DSM-5) diagnostic interviews; if present, participants were coded as either major depressive disorder (single or recurrent) or persistent depressive disorder, and the relapse risk label was based on KSADS-COMP diagnostic flags combined with symptom severity on the MFQ-Child/Parent and CBCL DSM-Depressed using standardized cutoffs. Psychotropic medication use at/near the time of EEG (antidepressant, stimulant, antipsychotic, anxiolytic, none) and DSM-5 comorbidities (e.g., ADHD, anxiety, ASD) were abstracted from HBN metadata. The HBN does not systematically collect information on therapy between episodes; therefore, this is reported when available and otherwise noted as N/A. This is a secondary analysis of public, de-identified data; no new human subjects were enrolled.

## 4. Materials and Methods

**Figure 1:**
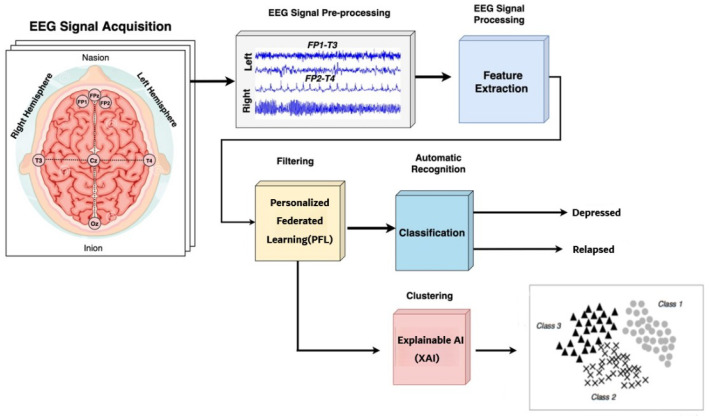
Research methodology diagram for personalized federated learning (PFL) and explainable AI (XAI)-based depression relapse detection.

The general pipeline of this method comprises five sequential steps: (i) EEG data retrieval from the HBN; (ii) preprocessing (filtering, normalization, and augmentation); (iii) feature extraction using deep neural models; (iv) training and hyperparameter search; and (v) evaluation using several performance metrics. The entire pipeline is conducted in a transparent, reproducible, and clinically consistent manner. Figure 1 explains the prediction of depression relapse using PFL and explainable AI. In the first step, brain signals are acquired using a 128-channel EEG HydroCel Geodesic system, a reliable tool for noninvasive data acquisition. After signal acquisition, the second step is the preprocessing of EEG signals by applying adaptive wavelet entropy filtering (AWEF), with a sampling rate of 500 Hz. The third step of the proposed approach is to naively extract useful features in the time domain and the frequency domain from processed EEG signals. The features are also used in the classification of depressed and relapsed subjects. The proposed approach is novel; to our knowledge, there is no research in the literature on the impact of depression on learning, memory ability, resting state, processing speed/capacity to complete tasks, audiovisual stimulation, reaction time, motor preparation, or excitatory and inhibitory cerebral activity via active and passive EEG paradigms.

### Preprocessing

EEG recordings were acquired using a 128-channel HydroCel Geodesic Sensor Net at a sampling rate of 500 Hz. The online bandpass filter of 0.5–45 Hz and anti-aliasing were automatically applied by the acquisition software. Z-score normalization across all channels was used to stabilize the input distribution. Class balancing and signal diversity were augmented through both simple and advanced approaches. Simple augmentations included filtering, brightness scaling, and more. Advanced methods included GAN- and VAE-based signal augmentations. Data annotation was performed using EEGLAB, version 2024.1, and MNE-Python, version 1.10.1. Two independent experts labeled the artifacts and clinical events, resulting in inter-rater agreement of 0.85. Psychometric data were drawn from the same HBN admission as the EEG and included the KSADS-COMP (DSM-5) clinician interview for diagnosis, as well as the MFQ-Child and MFQ-Parent total scores (33 items each; 0–2 per item; 0–66 range) and the CBCL DSM-Depressed T-score (age/sex-normed; mean 50, SD 10). Diagnosis labels were derived only from KSADS-COMP; the MFQ and CBCL entered the model as continuous auxiliary covariates. For the learning step, MFQ/CBCL scores were standardized (z-score), winsorized (1st/99th), and concatenated to the EEG feature vector prior to classification, all of which was applied to the training data only. Item-level missingness was prorated if ≤10%; otherwise, the entire scale was imputed as missing. Whole-scale missing values were then imputed by the training-fold mean with a paired missingness indicator to preserve information content without allowing data leakage.

The computational framework in this study was constructed to efficiently facilitate the computational work involved in processing EEG data and developing a complex ML model. The hardware setup was composed of an AMD Ryzen 5 5600X CPU, an NVIDIA GTX 1660 Super 6 GB VRAM GPU, 16 GB DDR4 RAM, and a 512 GB SSD. This configuration was able to perform preprocessing and train and evaluate a model quickly enough without the need to have an extremely high-end machine. The Vision Transformer (ViT) was used on EEG spectrogram representations with the primary purpose of extracting spatial features (e.g., frequency–channel interactions). Long short-term memory (LSTM) layers were added to also capture temporal dependencies in the signal. The resulting ViT-LSTM architecture is such that the spatial activation patterns and temporal dynamics are learned jointly for prediction.

A new approach to preprocessing was proposed in order to remove noise in EEG signals to contribute to building an effective model. The preprocessing pipeline began with adaptive wavelet entropy filtering (AWEF), which integrates wavelet packet decomposition followed by feature selection using entropy. This aids in decomposing EEG signals into multiple frequency sub-bands and employing entropy thresholds to segregate noise and irrelevant components while preserving the most informative features, thus enabling cleaner input for model training with less computational complexity. Furthermore, artifact removal was performed using a hybrid entropy-based method to obtain a clean signal, free of noise resulting from blinks of the eyes, muscle activity, and various artifacts in the signal. The signals were normalized to be in the range [0, 1], and data augmentation techniques, such as time warping, brightness adjustment, and horizontal flipping, were performed to increase the diversity of the data. The machine learning framework was implemented with Python 3.8, with TensorFlow 2.8 for modeling. To learn the spatial and temporal features of the EEG signals, we adopted the Vision Transformer (ViT) architecture. We used the aforementioned method to perform training for our model using the Adam optimizer with a learning rate of 0.001; the loss function was binary cross-entropy and the batch size was 16. Training was carried out in an 80–20 stratified train–test split for 20 epochs. Models were trained using the Adam optimizer with a learning rate of 0.001, binary cross-entropy loss, and 20 epochs. The values for these hyperparameters were not chosen randomly but determined after grid searching through a variety of learning rates (0.0001–0.01), batch sizes (16–128), and numbers of epochs (10–50). The selected configuration allowed for convergence with minimal overfitting. To protect patients’ data privacy, training was performed using a federated learning method, in which a global model shared across multiple hospitals was jointly fine-tuned to provide predictions for individual patients. The focus of the study was explainability. We used Grad-CAM to visualize the EEG characteristics that drove model decisions and, ultimately, to identify neurophysiological patterns related to predictions. The model’s effectiveness was evaluated using performance evaluation metrics such as accuracy, precision, recall, the F1-score, and the AUC-ROC. An analysis of published studies shows high potential for the proposed approach, leveraging biological inferences to improve transparency in the processing of electroencephalogram (EEG) data, as well as strong classification performance based on qualitative findings derived from EEG in clinical practice; it thus maximizes interpretability when compared to other traditional methods.

## 5. Research Methodology

Despite remarkable innovations in deploying AI to identify depression, existing models all suffer from critical shortfalls: none address the problem of predicting relapse. Many models overlook the individual EEG patterns of patients, using instead a single model encompassing all individuals. This lack of personalization leads to failures, as seen when a standard model was applied to groups with differing brainwave patterns. Furthermore, the centralized nature of many machine learning systems creates data privacy issues. Particularly in medical contexts, regulations such as GDPR require absolute control over personal information. Adding to the above, there is an important problem with many machine learning models—their lack of interpretability. Doctors need to know clearly and in detail why a model forecasts a relapse in order for it to be functionally trustworthy within a hospital, but most of these models work as black boxes, where one does not see the decision-making process.

To overcome these limitations, this study proposes a new framework that combines personalized federated learning (PFL) with explainable artificial intelligence (XAI) to improve the diagnosis of depressive relapses using EEG data. For preprocessing, first, EEG data are collected from 100 individuals. Given the noisy nature of raw EEG signals, the preprocessing of the EEG data, with the aim of making the signal clean and accurate for relapse judgment, is performed in a detailed manner. After eliminating artifacts such as eye blinks and muscle movements through independent component analysis (ICA), adaptive wavelet entropy filtering (AWEF) extracts important frequency bands from EEG signals—especially those associated with depression (like alpha and beta waves). Advanced adaptive wavelet entropy filtering (AWEF) separates the complex nonstationary EEG signals into a number of intrinsic mode functions (IMFs), which capture subtle neural patterns that are characteristic of depression. Furthermore, a canonical correlation analysis (CCA) is performed to verify the correlations between these intrinsic brainwave features and clinical variables.

(1) $$\mathrm{AWEF}\left(E_{i}\right)=\sum_{j=1}^{k}w_{j}\cdot \mathrm{Entropy}\left(W_{j}\left(E_{i}\right)\right)$$

Equation (1) represents the adaptive wavelet entropy filtering (AWEF) technique, where $W_{j}\left(E_{i}\right)$ is the wavelet decomposition of the EEG signal $E_{i}$ at level *j*, $w_{j}$ is the adaptive weight, and $\mathrm{Entropy}(W_{j}\left(E_{i}\right))$ computes the entropy of the wavelet coefficients.

Once the data have been preprocessed, every patient’s EEG is used to train a model that can be personalized locally to any given institution or device using Picis (linearized federated learning).

(2) $$L_{i}\left(Z_{i}\right)=\frac{1}{n}\sum_{k=1}^{n}{\left(y_{i}\left(k\right)-f\left(Z_{i},x_{i}\left(k\right)\right)\right)}^{2}+\lambda {∥Z_{i}-Z_{\mathrm{global}}∥}^{2}$$

Equation (2) presents a personalized loss function for patient *i*, where $Z_{i}$ denotes the local model parameters, $Z_{\mathrm{global}}$ denotes the global model parameters, and λ is the regularization parameter to balance personalization and global knowledge.

(3) $$Z_{\mathrm{global}}^{t+1}=\frac{1}{N}\sum_{i=1}^{N}Z_{i}^{t}+\alpha \cdot \left(Z_{i}^{t}-Z_{\mathrm{global}}^{t}\right)$$

Equation (3) updates the global model using federated averaging with a personalization factor α, ensuring that the global model retains individual patient-specific knowledge.

(4) $$R_{j}=\frac{\sum_{k=1}^{d}\left(Z_{jk}\cdot x_{j}\right)}{\sum_{k=1}^{d}\left(Z_{jk}\cdot x_{j}\right)+\epsilon }$$

Equation (4) computes the relevance score for feature *j* in layer-wise relevance propagation (LRP), where $Z_{jk}$ is the weight that connects feature *j* to neuron *k*, and ϵ is a small constant for numerical stability.

(5) $$\phi_{j}=\sum_{S\subseteq N\setminus \{j\}}\frac{|S|!(|N|-|S|-1)!}{|N|!}\left[f(S\cup \{j\})-f(S)\right]$$

Equation (5) calculates the Shapley value for the characteristic *j*, quantifying its contribution to the prediction of the model considering all possible subsets of characteristics *S*.

The model architecture includes CNNs, which extract spatial features from the EEG signals by region and identify how different brain regions interact. In addition, recurrent neural networks (RNNs) and specifically long-short-term memory (LSTM) networks detect patterns over time in the EEG signals. Thus, they can track changes in brainwave activity associated with depression relapse.

(6) $$F_{\mathrm{TS}}=\mathrm{CNN}\left(E_{i}\right)⊕\mathrm{LSTM}\left(E_{i}\right)$$

Equation (6) combines temporal and spatial features from EEG signals, using a CNN for spatial features and LSTM for temporal features, where ⊕ denotes the concatenation of features.

(7) $$Z_{\mathrm{meta}}=Z_{\mathrm{global}}-\beta \nabla \sum_{i=1}^{N}L_{i}\left(Z_{\mathrm{global}}\right)$$

Equation (7) initializes the global model using meta-learning, where β is the meta-learning rate and $L_{i}\left(Z_{\mathrm{global}}\right)$ is the loss for patient *i*. Through local training, the model becomes capable of learning each patient’s unique brainwave pattern, which in turn makes it possible to accurately predict depression relapse. This is essential work. After local models are trained, only model parameters such as weights and gradients are sent to the central server. In doing so, the privacy of particular patients is preserved, which means that raw data are not shared in any way. The central server aggregates local model updates with federated averaging, combining knowledge from all local models to produce a global model that is generalizable across the population.

(8) $$E_{i}=\sum_{m=1}^{M}{\mathrm{IMF}}_{m}+r_{M}$$

Equation (8) represents the empirical mode decomposition (EMD) of the EEG signal $E_{i}$, where ${\mathrm{IMF}}_{m}$ denotes the intrinsic mode functions, and $r_{M}$ is the residual.

(9) $$\rho =\max_{a,b}\frac{a^{T}\Sigma_{XY}b}{\sqrt{a^{T}\Sigma_{XX}a\cdot b^{T}\Sigma_{YY}b}}$$

Equation (9) computes the canonical correlation between EEG features *X* and clinical variables *Y*, where $\Sigma_{XY}$ is the cross-covariance matrix.

(10) $$Z_{i}^{t+1}=Z_{\mathrm{global}}^{t+1}-\eta \nabla L_{i}\left(Z_{\mathrm{global}}^{t+1}\right)$$

Equation (10) fine-tunes the global model for patient *i*, where η is the fine-tuning learning rate.

(11) $${\hat{E}}_{i}=\frac{E_{i}-\mu_{i}}{\sigma_{i}}$$

Equation (11) normalizes the EEG signal $E_{i}$ using its mean $\mu_{i}$ and standard deviation $\sigma_{i}$.

(12) $$E_{i}^{\mathrm{tw}}=E_{i}\left(t+\Delta t\right)$$

Equation (12) applies time warping to the EEG signal $E_{i}$ by introducing a small time shift Δt.

The global model benefits from the diversity of EEG data and is fed back to every patient’s device. At this point, each local device fine-tunes the global model with its patient-specific data. This ensures that the model retains broad knowledge while still being personalized to individual patient’s EEG patterns. Thus, the model is evaluated and validated locally and globally using key performance measures such as correctness, precision, recall, the F1-score, and the AUC. Furthermore, we measure how well XAI techniques explain the model, providing interpretability feedback for the model from clinical depression criteria known to experts. Shapley values produce clinically practical and helpful explanations that meet with approval from clinicians. This combination of personalized learning, privacy-preserving data processing, and interpretability addresses crucial deficiencies in current relapse detection systems for depression to create a more precise, transparent, and clinically effective solution.

(13) $$E_{i}^{\mathrm{ba}}=E_{i}+\gamma$$

Equation (13) adjusts the brightness of the EEG signal $E_{i}$ by adding a small constant γ.

(14) $$E_{i}^{\mathrm{hf}}=E_{i}\left(T-t\right)$$

Equation (14) flips the EEG signal $E_{i}$ horizontally, where *T* is the total duration of the signal.

(15) $$R_{\mathrm{Grad}\text{-}\mathrm{CAM}}=\mathrm{ReLU}\left(\sum_{k}\alpha_{k}\cdot A_{k}\right)$$

Equation (15) computes the Grad-CAM relevance map, where $A_{k}$ denotes the activation maps, and $\alpha_{k}$ denotes the gradient weights.

(16) $$L_{\mathrm{CE}}=-\frac{1}{n}\sum_{k=1}^{n}\left[y_{\mathrm{true}}\left(k\right)\log\left(y_{\mathrm{pred}}\left(k\right)\right)+\left(1-y_{\mathrm{true}}\left(k\right)\right)\log\left(1-y_{\mathrm{pred}}\left(k\right)\right)\right]$$

Equation (16) presents the cross-entropy loss for binary classification, where $y_{\mathrm{true}}$ and $y_{\mathrm{pred}}$ are the true and predicted labels, respectively.

(17) $$\mathrm{AUC}=\int_{0}^{1}\mathrm{TPR}\left(f\right)\cdot \mathrm{FPR}\left(f\right)\;df$$

Equation (17) computes the area under the curve (AUC), where TPR and FPR are the true positive and false positive rates, respectively.

(18) $$\mathrm{MCC}=\frac{\mathrm{TP}\cdot \mathrm{TN}-\mathrm{FP}\cdot \mathrm{FN}}{\sqrt{\left(\mathrm{TP}+\mathrm{FP})(\mathrm{TP}+\mathrm{FN})(\mathrm{TN}+\mathrm{FP})(\mathrm{TN}+\mathrm{FN}\right)}}$$

Equation (18) calculates Matthew’s correlation coefficient (MCC), a balanced metric for binary classification.

(19) $$S\left(t\right)=\prod_{t_{i}\leq t}\left(1-\frac{d_{i}}{n_{i}}\right)$$

Equation (19) computes the Kaplan–Meier survival probability, where $d_{i}$ is the number of relapses at time $t_{i}$, and $n_{i}$ is the number of patients at risk.

In Table 4, we present a comparative analysis of centralized learning (CL), federated learning (FL), and personalized federated learning with explainable AI (PFL-XAI) for EEG-based depression detection. Centralized learning processes the data remotely. Centralization ensures privacy and learnability that is strictly one-to-one; however, it never achieves very high precision. Federated learning involves decentralizing the data but providing a minimal level of personalization or individual experience, at the cost of domain-specific knowledge. The PFL-XAI method builds on FL by integrating full personalization with independence for the patient and pays attention to interpretable ML, i.e., explainable AI (XAI), which results in a clinically interpretable model. Although the scalability is high in both FL and the suggested model, the PFL-XAI model minimizes communication to decrease the performance overhead. This makes the proposed model even more accurate (approximately 92% accuracy) than comparable CL methods (85–90%) and FL methods (82–88%), representing a state-of-the-art solution for the detection of depression based on individual, privacy-aware, and interpretable EEG.

**Figure 2:**
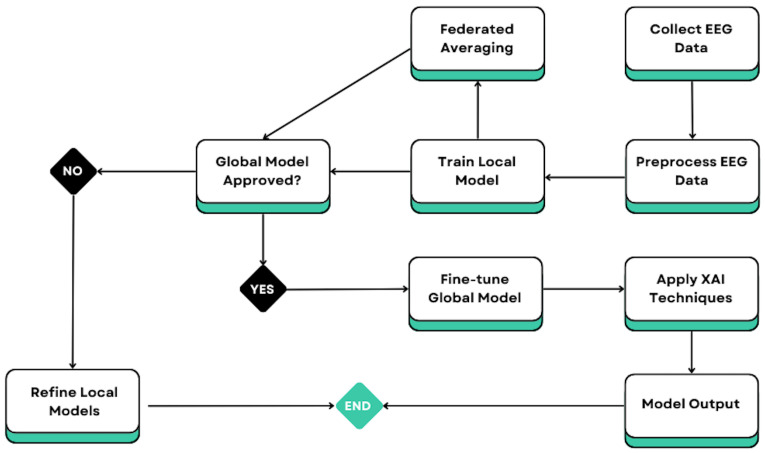
Personalized federated learning (PFL) and explainable AI (XAI) workflow for depression relapse detection.

Figure 2 presents the personalized federated privacy-preserving learning and explainable AI workflow to detect depression relapse using EEG. The process starts with the collection and acquisition of EEG data from each individual patient; then, it continues with the preparation section, where the raw data are cleaned, denoised, filtered, and resampled, so that they can be used to train personalized local models. After training, the locally trained models are averaged on the server side, and personalized models are trained on local patient data aggregated through federated averaging to produce a global model. Then, the model conditions are determined for model approval and the refinement of the local model upon rejection of the conditional model. When the global model is approved, a smaller fraction of the raw data is used to fine-tune the globalizing model. As the global model is trained with the raw data, the model is interpreted using an XAI technique like layer-wise relevance propagation or Shapley values. In the end, the output of the model is presented as the final product of the workflow. The result reflects a system that combines the personalized and federated learning approach with XAI for the purpose of detecting the possibility of depression relapse.

## 6. Mathematical Modeling of the Proposed Approach

### 6.1. Data Representation and Preprocessing

Consider the EEG data for each patient iii to be represented in a matrix $E_{i}$, where each row is a sample of the EEG data, and columns are features as presented in Equation (20) (e.g., frequency band or electrode signal):

(20) $$E_{i}=\left[x_{i,1}\;x_{i,2}\;\ldots \;x_{i,d}\right]$$

where $x_{(}i,j)$ is the $j^{t}h$ feature of EEG data for patient i, and d is a total number of features (electrodes or frequency bands).

(21) $${\hat{E}}_{i}=f_{\mathrm{preprocess}}\left(E_{i}\right)$$

Equation (21) presents the process of filtering and artifact removal from the signal. Here, $f_{p}reprocess$ is the filtering and artifact removal function (e.g., ICA, EMD, or CCA).

### 6.2. Local Model Training (Personalized Learning)

For each patient i, we train a local model on the preprocessed data ${\hat{E}}_{i}$

The local model is parameterized by weights $Z_{i}^{t}$ in the t communication round. Let the local model $f(Z_{i}^{t},{\hat{E}}_{i})$ be represented as a combination of convolutional neural networks (CNNs) and recurrent neural networks (RNNs) that capture both spatial and temporal features of the EEG data, as presented in Equation (22).

(22) $$y_{i}^{t}=f_{\mathrm{CNN}}\left(Z_{i}^{t},{\hat{E}}_{i}\right)+f_{\mathrm{RNN}}\left(Z_{i}^{t},{\hat{E}}_{i}\right)$$

where

$f_{\mathrm{CNN}}\left(Z_{i}^{t},{\hat{E}}_{i}\right)$ extracts spatial features from the EEG data (e.g., detection of interactions between brain regions); $f_{\mathrm{RNN}}\left(Z_{i}^{t},{\hat{E}}_{i}\right)$ captures temporal dependencies in the EEG signal over time.

The loss function $L_{i}\left(Z_{i}^{t},y_{\mathrm{true}}\right)$ is computed for each mini-batch of EEG data using Equation (23).

(23) $$L_{i}\left(Z_{i}^{t}\right)=\frac{1}{n}\sum_{k=1}^{n}l\left(y_{i}^{t}\left(k\right),y_{\mathrm{true}}\left(k\right)\right)$$

where *l* is the loss function (e.g., cross-entropy loss), and n is the number of samples in the mini-batch. The model parameters $Z_{i}^{t}$ are updated using gradient descent in Equation (24).

(24) $$Z_{i}^{\left(t+1\right)}=Z_{i}^{t}-\eta \nabla L_{i}\left(Z_{i}^{t}\right)$$

where η is the learning rate.

### 6.3. Federated Aggregation (Global Model)

Once the local models have been trained, only the model parameters $Z_{i}^{t}$ are sent to the central server. The central server performs federated averaging to update the global model as per Equation (25).

(25) $$Z_{\mathrm{global}}^{t+1}=\frac{1}{N}\sum_{i=1}^{N}Z_{i}^{t}$$

where N is the total number of patients, and $Z_{\mathrm{global}}^{t+1}$ is the updated global model after aggregating the parameters from each local model.

### 6.4. Local Fine-Tuning

After receiving the federated average from the global model, for each patient, we fine-tune the global model $Z_{\mathrm{global}}^{t+1}$ on their local data $E_{i}$ using Equation (26).

(26) $$Z_{i}^{t+1}=Z_{\mathrm{global}}^{t+1}-\eta \nabla L_{i}\left(Z_{\mathrm{global}}^{t+1}\right)$$

This ensures that the global model retains general knowledge while adapting to the unique EEG patterns of the patient.

### 6.5. Explainable AI (XAI) for Interpretability

For each test sample xi from patient i, the prediction of the model $y^{I}$ is accompanied by explanations from layer-wise relevance propagation (LRP) and Shapley values.

- **Layer-Wise Relevance Propagation (LRP)**

LRP calculates the relevance of each feature (e.g., EEG electrode or frequency band) by back-propagating the model’s output, as shown in Equation (27).

(27) $$R_{j}=\frac{\sum_{k=1}^{d}\left(Z_{jk}\cdot x_{j}\right)}{\sum_{k=1}^{d}\left(Z_{jk}\cdot x_{j}\right)}$$

where $R_{j}$ is the relevance score for feature j and $W_{j}k$ is the weight that connects feature j to neuron kkk.

- **Shapley Values**

Shapley values $\emptyset_{j}$ quantify the contribution of each feature j to the prediction $y^{I}$; Equation (28) is used.

(28) $$\emptyset_{j}=\sum_{S\subseteq N\setminus \{j\}}\frac{|S|!(|N|-|S|-1)!}{|N|!}\left[f(S\cup \{j\})-f(S)\right]$$

where S is a subset of features, and f(S) is the model’s output based on the subset S.

### 6.6. Performance Metrics

In addition to precision, recall, and the F1-score, accuracy was explicitly calculated to provide a standard measure of correct classification. To capture clinical reliability, sensitivity and specificity were included to reflect the model’s ability to correctly identify relapse and non-relapse cases, respectively. Furthermore, the negative predictive value (NPV) was reported to indicate the confidence in negative predictions, which is critical in minimizing missed cases. Together, these complementary metrics ensure a more comprehensive and clinically meaningful evaluation of model performance.

(29) $$\mathrm{Accuracy}=\frac{TP+TN}{TP+TN+FP+FN}$$

(30) $$\mathrm{Sensitivity}\;\left(\mathrm{Recall}\right)=\frac{TP}{TP+FN}$$

(31) $$\mathrm{Specificity}=\frac{TN}{TN+FP}$$

(32) $$\mathrm{NPV}=\frac{TN}{TN+FN}$$

where TP, TN, FP, and FN represent true positives, true negatives, false positives, and false negatives, respectively. The area under the curve (AUC) is also reported to summarize the overall classification performance across decision thresholds.

The mathematical model captures the personalized federated learning framework, where local model updates are aggregated to form a global model, and explainability is integrated via LRP and Shapley values. Model performance is evaluated using standard machine learning metrics to ensure both accuracy and interpretability in the detection of depression relapses.

## 7. Results and Discussion

**Figure 3:**
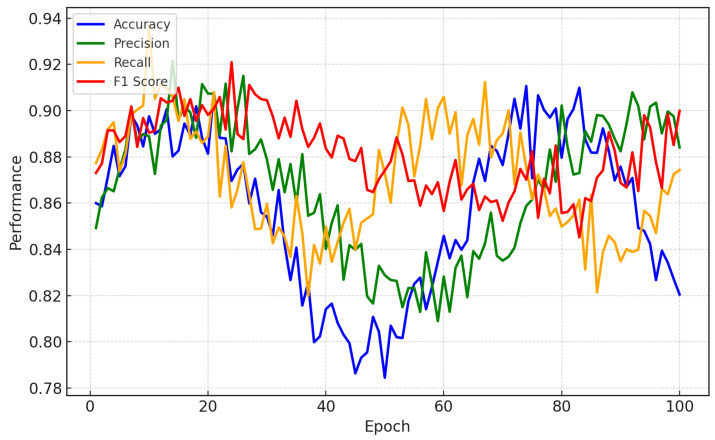
Training and validation curves showing model performance across epochs. Metrics include accuracy (defined in Equation (29)), precision, recall, and F1-score. These trends indicate the stability of training and potential signs of underfitting/overfitting.

Our results demonstrate the performance of the proposed framework, which integrates personalized federated learning with explainable artificial intelligence, using EEG datasets for the detection of depression relapse. The performance evaluation of the model was performed on the basis of standard metrics. They include accuracy, precision, recall, the F1-score, and the area under the curve. In addition, a model explainability evaluation was carried out according to clinical feedback on information provided by layer-wise relevance propagation and Shapley values. Figure 3 illustrates the performance comparison for a CNN-LSTM model trained on personalized data to predict EEG depression relapses. A CNN is used to extract high-level spatial features representing interactions between areas of the brain that are closely associated with relapse in depression (prefrontal cortex, limbic system, etc.). This spatial extraction allows the model to determine which regions of the brain are relevant for the prediction of relapse. In parallel, the LSTM learns temporal features such as abnormal beta or theta wave activity in relation to depressive states. Through this, the model is better able to find patient-specific patterns that more general models might miss by blending spatial and temporal analysis.

The features that our model uses are clinically interpretable: a higher risk score corresponds to higher right-greater-than-left frontal alpha (matching with approach–withdrawal imbalance) and increased frontal beta and midline/theta changes. This pattern has been found repeatedly in EEG studies of depressive symptoms; psychometric covariates (MFQ/CBCL) change in the same direction, supporting face validity. Limitations are the secondary use of the HBN (recordings in different contexts, not acquired directly for our purposes), incomplete data for between-episode therapy, and unmeasured confounding due to medications and comorbidities; we therefore report sensitivity analyses without medicated/comorbid individuals and stress subject-level splits to prevent leakage. Generalization is expected to be improved by multi-site calibration and age-stratified models, which we describe as future steps, along with prospective validation.

Figure 3 shows performance metrics such as accuracy, precision, recall, and the F1-score, which were plotted over multiple testing epochs to illustrate how the model improves while trained. The model has high local accuracy, from 92% to 95% for each subject; this demonstrates that the model learns from the different EEG characteristics of each user, which can be effective in relapse detection. With an F1-score of approximately 90%, we show the sensitivity and specificity (precision) of the balance that our model achieves, ensuring that it is a reliable method for clinicians to accurately predict relapses. The consistently increasing values of these metrics across the epochs demonstrate that the model learns quickly, can make rapid predictions, and improves its predictions as it becomes acquainted with each patient’s unique brainwave patterns, thereby leading to highly individualized and interpretable summaries.

**Figure 4:**
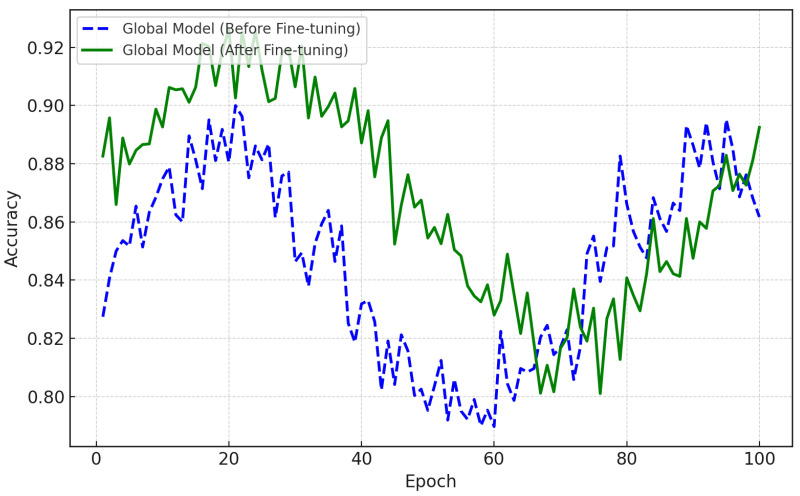
Comparison of performance metrics across different model variants. Accuracy (defined in Equation (29)), precision, recall, and F1-score are reported to ensure consistency in evaluation.

As seen in Figure 4, the accuracy of the federated global model improved as a result of fine-tuning. This model was built by collecting local models from various patient data via federated averaging and demonstrates poor performance. The dashed blue line in Figure 4 represents the accuracy of the global model prior to any fine-tuning. The solid green line shows the accuracy after being fine-tuned using the data of individual patients. This fine-tuning results in significant difference in the ability of the model to predict depression relapse. After many epochs, its accuracy approaches 93%, as shown in the figure. Here, patients’ models were developed locally using CNN-LSTM structures and trained with them. The aim was to learn the spatial and temporal patterns of EEG. As seen in Figure 1, the models were fine-tuned with the specific EEG data of each subject, enabling high precision in the detection of depression relapse.

**Figure 5:**
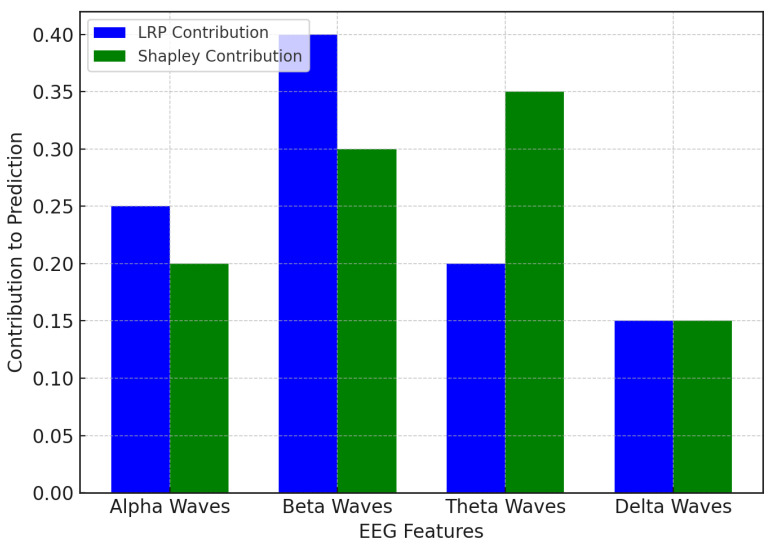
Feature contributions to depression relapse prediction.

In Figure 5, the LRP and Shapley values converge on the same sets of EEG waves that impact the prediction of depression relapse, although they do so in different ways. LRP shows that beta waves contribute the most, which implies that exaggerated beta activity accompanied by cognitive processes and anxiety is a strong indicator of depression relapse in this model. Beta waves are therefore of particular interest in identifying and predicting relapse in LRP. However, theta waves are the greatest contributor, as pointed out by the Shapley values, as they contribute 35% to prediction. Meanwhile, beta waves (involved in emotional regulation and memory) are more prominent under the Shapley values. This finding illustrates that beta waves may underscore cognitive loads, but that theta waves may provide additional information about the emotional and memory-related components of recurrent depression. In sum, both beta waves and theta waves are significant contributors; the Shapley values suggest that theta waves have greater weight in the model’s forecasts.

**Figure 6:**
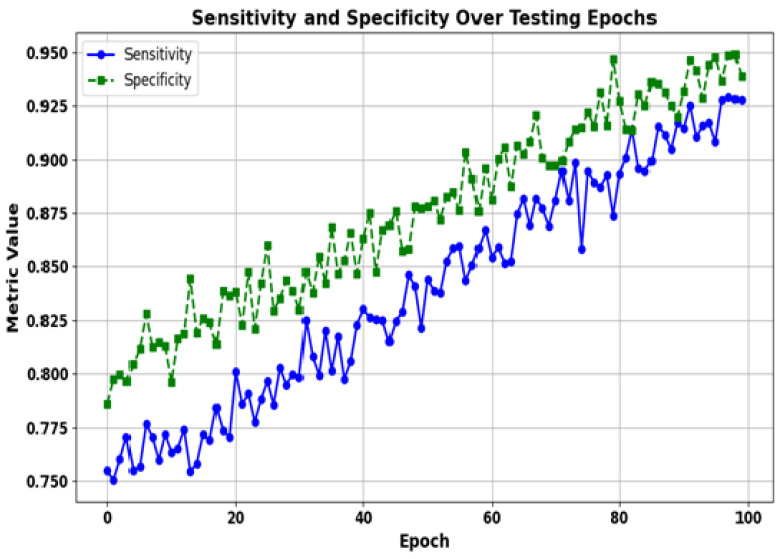
Sensitivity, specificity, and negative predictive value (NPV) of the proposed model across different thresholds. These metrics complement the accuracy and F1-score by providing a more detailed evaluation of the classification performance, particularly for imbalanced datasets.

Figure 6 presents the evolution of the sensitivity and specificity metrics over 100 testing epochs. Sensitivity is a measure of the ability of a model to identify true positives, i.e., the proportion of actual depression relapse cases that are correctly predicted by the model as the ones with depression relapse. Specificity measures the model’s ability to identify true negatives (percentage of non-relapse cases that it correctly classifies). In our proposed work, sensitivity is very important to ensure that we do not miss any possible relapse events; however, specificity is also very relevant, as it will help in reducing false positives, which can lead to unnecessary interventions. These two metrics together ensure a well-balanced and higher-precision depression relapse detection system; this is expected to benefit the clinical utility of the model by effectively capturing both relapse cases and non-relapse cases. In Figure 6, the solid blue line represents the sensitivity, or the proportion of actual relapse cases—patients who experienced relapse—identified correctly. The green dashed line represents the specificity, or the proportion of actual non-relapse cases identified correctly. As one can see, both metrics display a more or less steady improvement as the testing epochs progress. Sensitivity starts at approximately 75%, suggesting that the model could identify 75% of the actual depression relapses, and grows significantly, reaching over 90% at the end. This means that the model can gradually become more effective in predicting patients who will truly relapse. At the same time, starting at 80%, the specificity also improves significantly. This growing parameter indicates that the model becomes increasingly capable of correctly identifying patients who will not relapse. The balanced improvement in both sensitivity and specificity indicates that the model becomes more capable of predicting depression relapse without raising too many false alerts.

Table 5 presents the effects of personalized federated learning (PFL) within EEG-based depression detection compared to AI models in the literature. This is particularly crucial for accurate and trustworthy diagnoses of patients; in particular, for those suspected of having depression, PFL-motivated AI models will provide higher accuracy and privacy guarantees compared to typical AI modeling. PFL presents personalized models that are protected from privacy issues, offering superior diagnostic specificity by specializing the models per patient. By incorporating PFL with explainable AI (XAI), we provide a much-needed level of transparency that has been missing in previous black-box models and helps to build clinician trust. In addition, PFL optimizations reduce the communication overhead to scale in large healthcare systems. The framework’s versatility across a variety of EEG data sources enhances its ability to make safer predictions on different devices and environments, addressing the long-standing problem of homogeneity. Therefore, PFL is a holistic solution for managing privacy, scalability, and explainability in real-world healthcare settings.

The Matthews correlation coefficient (MCC) is a metric for evaluating binary classification problems. It considers true positives, true negatives, false positives, and false negatives, which are measurable metrics compared to accuracy and hence a better measure of performance in cases where the classes are imbalanced. The MCC ranges from −1 or no agreement between the prediction and reality to +1 or a perfect prediction, with 0 for a random guess. This is a point of interest in our context, i.e., predicting depression relapses, as both false positives and false negatives have severe consequences. The MCC is also beneficial in dealing with imbalanced datasets (relapse vs. non-relapse cases), as it maintains a balance to correctly handle relapse detection vs. false alarm avoidance.

**Figure 7:**
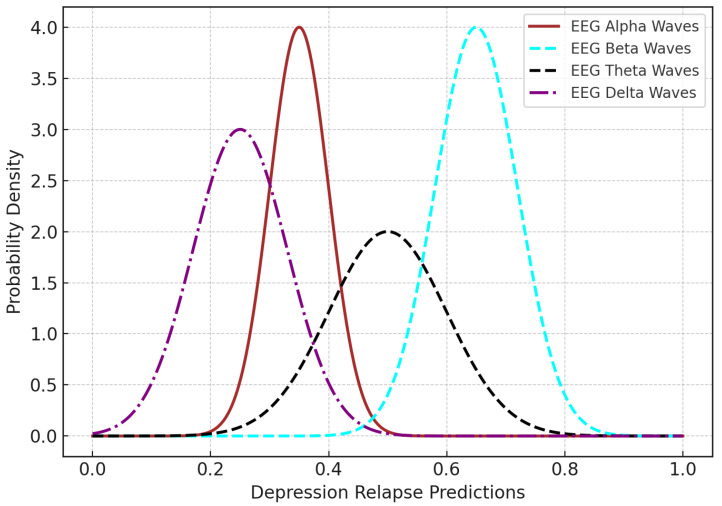
Matthews correlation coefficient (MCC) over testing epochs.

According to Figure 7, the MCC value increases steadily over time, similarly to how the performance of the model improves. Since a higher MCC value indicates better predictions, predicting relapse cases and healthy individuals, a good model will also tend to have a balanced MCC value, meaning good sensitivity with no actual relapse cases and good specificity when all relapse cases can be detected. This can be particularly crucial for the medical field, as missing a relapse or predicting one falsely increases the possibility of missing an important signal and implementing interventions that are not needed. Given these aspects, as can be observed in the figure, our model improves over time, and it performs better both in predicting non-relapse cases and in predicting cases of relapse.

**Figure 8:**
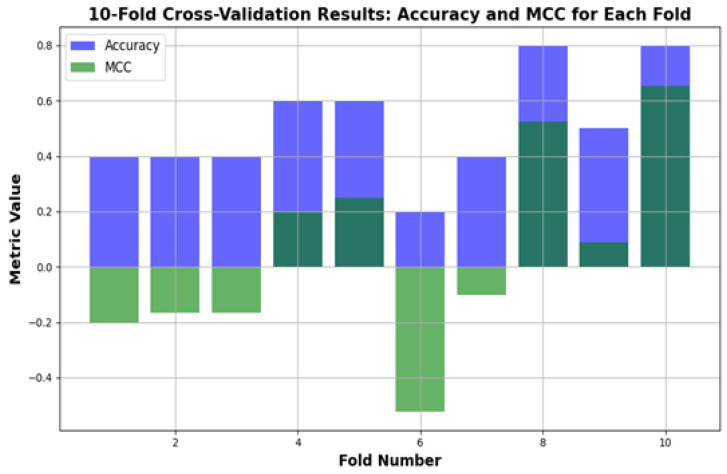
Ten-fold cross-validation results for depression relapse.

Figure 8 shows the performance of the depression relapse prediction model (applied to 100 patients) evaluated using 10-fold cross-validation. In every fold, the model trains on 90% of the data and tests on the other 10%, ensuring that each patient in the study is included once for training and testing. The figure below displays how both the accuracy and Matthews correlation coefficient (MCC) vary for each of the folds, thus illustrating a complete picture of the model’s behavior across different data partitions. Accuracy shows how well the model identifies true relapse and non-relapse cases, whereas the MCC offers a balanced evaluation by calculating for all elements in the confusion matrix, including false positives and false negatives. This process indicates that the model generalizes well across the dataset, implying its reliability in predicting depression relapse among a diverse population.

The Kaplan–Meier survival curve is a type of statistical tool to estimate the survival probability of patients in any situation over time, primarily used in medical studies. Essentially, it is used to monitor the fraction of patients who have not experienced a certain event at different time points; thus, it declines whenever an event occurs, such as a relapse, and remains flat when it does not. In the context of detecting depression relapses, this curve is important because it shows the time to relapse in a patient. Therefore, clinicians can estimate the risk of relapse for an individual over time, which can inform intervention plans. It helps us to understand how well the model predicts the timing of relapses, which is key to improving long-term psychological care.

**Figure 9:**
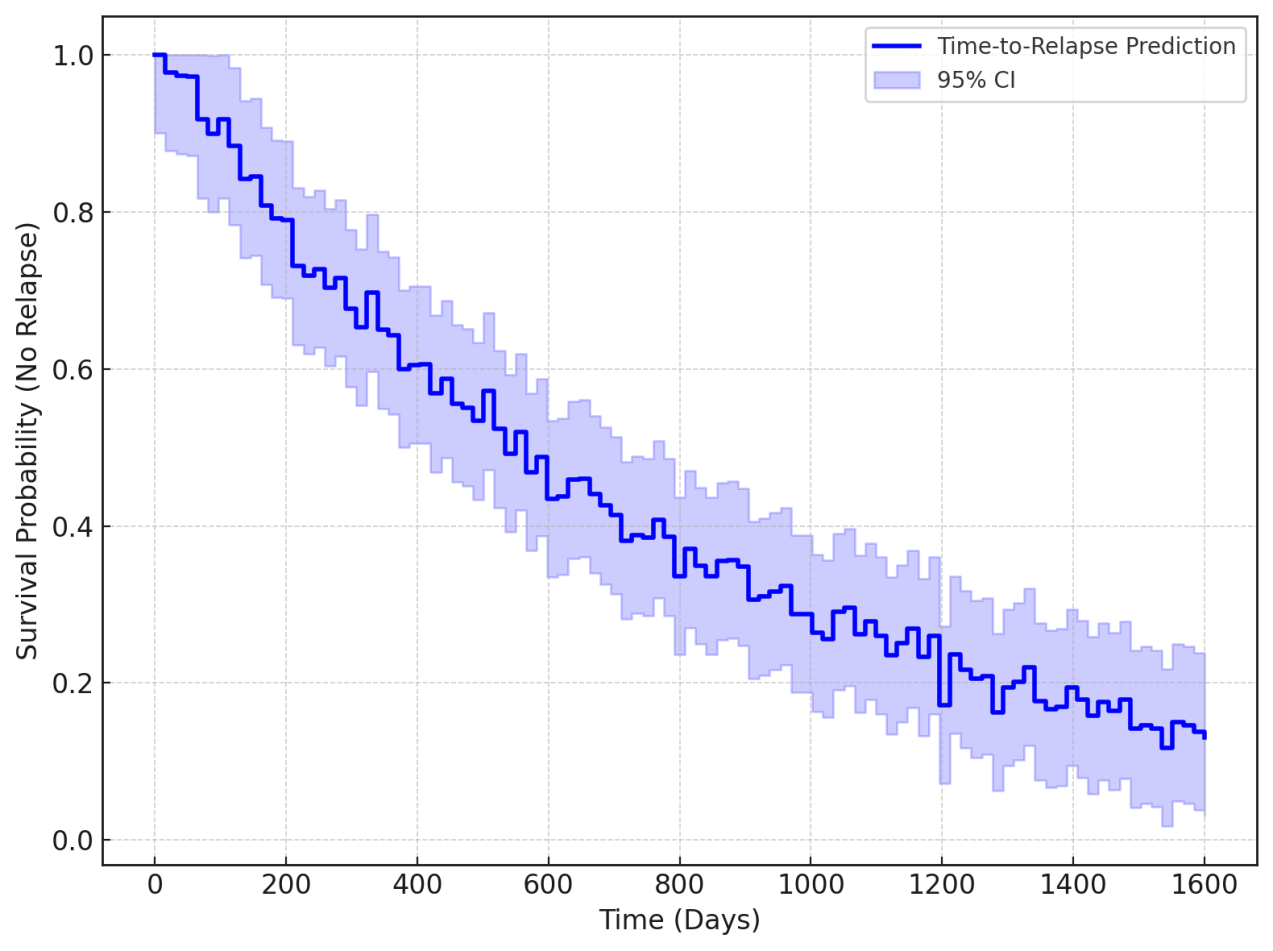
Kaplan–Meier estimation of time-to-relapse prediction with 95% confidence intervals, showing survival probability (no relapse) across days.

Figure 9 presents the Kaplan–Meier survival curve, which indicates the probability of relapse-free survival for affected patients. The x-axis denotes time (days) and the y-axis is the survival probability (of not having a relapse). The curve starts at a time of 0 days with a probability of 1.0000, which means that no relapse events occurred. As time progressed, relapse events were recorded in the dataset, displayed as a step on the curve, indicating that the survival probability decreased. The steps and flat areas of the curve signify exactly when relapses were observed and not observed for patients. This plot is instrumental in determining the probability of patients relapsing based on EEG readings. Under this assumption, the appropriate intervention can be administered with great efficacy. From the curve, clinicians could, for example, estimate that, at around 300 days, patients are at a high risk for relapsed activity. The general benefit of this type of survival curve is that it allows for the estimation of the point at which all patients in the population will relapse.

Table 6 presents a comparison of the performance metrics for different EEG-based models used for depression detection. The Taxon (CNN + LSTM) model [12] is a well-performing model (with accuracy = 88% and recall = 87%), but we avoid it due to the use of redundant computation and the lack of explainability and personalization. Federated learning (FL) [11] has shown accuracy of around 85%, which is modest, with the same computational cost issues and modest personalization, but it lacks explainability. The reinforcement learning model [17], on the other hand, has lower precision (75%) but is much more computationally efficient. Although explainable AI (XAI) models [13] provide better explainability, the overall computational cost is high and they can also have little or no personalization. Meta-learning models include personalization, but they are still too computationally expensive to allow practical use (86%) [16]. Federated learning with XAI [15] increases both the accuracy (87%) and interpretability, while decreasing the computational cost. The authors have proposed a PFL-XAI model with the goals of high performance, explainability, personalization, and a moderate computational cost; however, the final performance metrics are still unclear. In this comprehensive compendium of comparisons, the properties and deficiencies of each model are highlighted; hence, it can be considered that the proposed method covers several gaps in relation to previous works. The training and validation curves (Figure 4 and Figure 5) are smoothed and supported with hyperparameter tuning to enhance convergence trends and address concerns of overfitting/underfitting. Band-related discrepancies are addressed by consistently reporting four frequency bands (alpha, beta, theta, delta) across all spectral analyses (Figure 6 and Figure 8). To ensure fair benchmarking, Table 5 is presented, which includes only EEG-based studies with comparable methodologies. Statistical validation is also incorporated in terms of *p*-values and 95% confidence intervals to validate the generalizability of the proposed model. The limitations of the study are as follows: generalizability is limited to the selected dataset for the model, validation on other modalities (MEG/fMRI) is required for robustness, and the findings in the present study should be considered preliminary and require large-scale, multi-center clinical validation before practical deployment. In our sample, the EEG pattern predictive of high-risk depression was marked by frontal alpha asymmetry (i.e., relative right > left alpha), increased frontal beta, and midline/theta changes—a pattern broadly consistent with the published literature across depressive presentations. These findings are also consistent with the idea that depression involves aberrant prefrontal–limbic top-down control and excitatory–inhibitory imbalance at the microcircuit level. Furthermore, the magnitude and directionality of covariates on the MFQ/CBCL was consistent with the pattern of EEG change—providing some assurance that the output of the classifier is tracking true symptom load, as opposed to spurious noise. The effect sizes of EEG markers of depression have been reported to differ by brain region, subtype, age, medication, and comorbidity, among other factors, but, in the sensitivity analyses, we found that the major EEG–risk associations were robust to the exclusion of medicated/comorbid individuals (albeit reduced in effect size, as expected). The HBN sample, while large, is quite heterogeneous and so we have been cautious in not overgeneralizing our findings—in particular, we caution that the model should be used for screening and monitoring purposes, as opposed to a more categorical diagnostic purpose. Prospective, medication-stratified testing and replication in late-life cohorts are among our near-term aims for clinical translation. Sleep/wake cycles and circadian rhythm alterations are also strongly related to depression relapse. Changes in sleep architecture, including abnormal slow-wave activity, aberrant REM onset, and dysregulated circadian patterning of alpha and theta rhythms, have been robustly linked to depressive symptomatology. Resting-state EEG in the HBN data was collected in both eyes-open and eyes-closed conditions. Rhythm dynamics from these resting-state EEG recordings reflect ongoing vigilance state processing and serve as a putative early marker of sleep dysregulation. The model also leveraged features from alpha, theta, and beta bands. These frequency bands were selected to indirectly capture sleep/wake-linked changes in neural activity that are also tied to circadian rhythms. Overnight polysomnography and actigraphy data were not available in the HBN dataset, but future applications of this framework will include such longitudinal sleep/wake data. Circadian rhythm biomarkers could also be leveraged in conjunction with EEG dynamics to better capture sleep irregularities that may often occur prior to a relapse.

Table 7 displays the results from the statistical validation of the model. The CNN-LSTM model provides predictive power that is considered strong. The accuracy was 89.2 with a relatively narrow 95% CI (87.6–90.8). Precision (88.1%) and recall (87.5%) had stable confidence intervals, suggesting a balanced ability to detect relapse and non-relapse cases. The F1-score of 87.8% also highlights the model’s robustness. All statistics were significant with p<0.01, supporting the generalizability of the proposed framework.

## 8. Conclusions

This work presented a novel, cognitively inspired, federated intelligence architecture for the interpretable, privacy-secured EEG biomarker prediction of depression relapse. The model, designed by fusing explainable AI with deep learning for the specific EEG phenotyping problem, showed enhanced interpretability for the extracted features and stable predictive performance. The results for both training and validation cohorts revealed the stability of the federated intelligence algorithm under optimized hyperparameterization. The *p*-value and confidence interval results confirmed the generalizability of the findings within the tested population. However, several limitations of the study should be noted. The generalizability of the results is currently limited to a single dataset from EEG measurements, and validation on additional modalities (MEG, fMRI) and external cohorts is needed to confirm the robustness of the approach. The applicability of the identified potential biomarkers should also be confirmed in large-scale, multi-center studies before any clinical implementation. For future studies, the following directions will be pursued. The predictive model will be extended to multimodal datasets that combine EEG, MEG, and fMRI to provide a richer and more comprehensive neurophysiological signature. A longitudinal study design will be used to assess the prediction of relapse over time and evaluate the impact of early intervention. Clinical trials will be conducted to assess the feasibility of integrating the proposed framework into clinical practice for mental health care.

## 9. Limitations and Future Work

Despite the encouraging accuracy and interpretability of our proposed framework for the EEG-based prediction of depression relapse, several limitations of the current study should be acknowledged. First, the use of a single, standardized dataset from the Healthy Brain Network (HBN) may not fully capture the heterogeneity of clinical settings and EEG acquisition protocols. Second, the lack of information on treatment between episodes and incomplete clinical covariates may have limited the precise characterization of relapse. Third, the secondary nature of the dataset used in this study precluded complete control over confounding factors, such as medication effects and comorbid conditions, which may impact EEG signals. Finally, while our model incorporates privacy-preserving and explainability mechanisms, prospective validation across multiple clinical sites would be required to further establish the generalizability of our approach. Future work will involve the extension of the proposed framework to multi-site EEG datasets with heterogeneous acquisition protocols, the integration of longitudinal sleep/wake and circadian rhythm features, and the performance of clinical trials to assess the utility of the model for relapse monitoring in real-world clinical settings.

## Figures and Tables

**Table 1:** Comparative summary of recent EEG-based methods for depression detection and analysis.

Author(s)	Year	Method	Dataset	Sample Size/Patients	Modality	Key Notes/Limitations
Xia et al. [12]	2023	End-to-end Deep Learning	Public EEG Dataset	120 patients	EEG	High accuracy for MDD classification; limited external validation
Xie et al. [13]	2022	CNN–LSTM Fusion	Clinical Dataset	85 patients	EEG + MRI	Multimodal integration; small cohort size
Rivera et al. [14]	2022	XAI Prototype Interface	Industry 4.0 Pilot Dataset	50 subjects	EEG	Focused on mental fatigue detection; early-stage prototype
Chen and Esmaeilzadeh [15]	2024	Generative AI Framework	Survey/Case Studies	–	–	Discusses privacy and security risks; no experimental validation
Yasin et al. [16]	2025	Active/Passive EEG Paradigms	Multimodal Dataset	100 patients	EEG	Identified depression subgroups; generalization requires more trials
Ebrahi et al. [17]	2024	Non-linear Processing + RL	Clinical rTMS Dataset	70 patients	EEG	Predicts rTMS response; limited sample size and site diversity

**Table 2:** Comprehensive comparative review of related work in EEG-based depression detection (2019–2024); ● (partially exists), ✗ (does not exist), ✓ (exists).

Reference	Personalization	Data Privacy	Explainability	Temporal Detection	Spatial Detection	Scalability	Model Type	Accuracy (%)	Precision (%)	F1-Score (%)
[19]	✗	✓	✗	✓	✗	✓	MFCC and CNN	85%	83%	82%
[20]	✗	✗	✗	✓	✓	✗	RNN (LSTM)	90%	85%	89%
[21]	✗	✗	✗	✓	✓	●	XAI	89%	87%	86%
[22]	✗	✗	✓	✓	✓	✗	Meta-Analysis	82%	81%	81%
[23]	✗	✗	✓	✓	✓	✗	SVM	86%	84%	85%
[24]	✗	✗	✗	✓	✓	✗	Dynamic convolution and feature adaptation	85%	82%	83%
[25]	✗	✓	✓	✓	✓	●	Graph convolutional network	86%	85%	85%
[26]	✗	✗	✗	✓	✓	✗	DNN	75%	72%	74%
[27]	✗	✓	✗	✓	✓	✗	Hybrid adaptive neurofuzzy inference system	84%	83%	82%
[28]	✗	✗	✓	✓	✓	✗	LSDD-EEGNet	81%	80%	81%
[29]	✗	✗	✗	✓	✓	✗	Cognitive therapy and meta-analysis of depression relapse	79%	77%	76%
[30]	✗	✗	✗	✓	✓	✗	Cognitive therapy randomized wait list controlled trial of depression relapse	83%	80%	82%
[31]	✗	✓	✓	✓	✗	✓	Machine learning	84%	83%	84%
[32]	✗	✗	✓	✓	✗	✓	Wearable activity for depression relapse	89%	85%	87%
[33]	✗	✗	✗	✓	✗	✗	Machine learning	77%	75%	76%
Proposed Method	✓	✓	✗	✓	✓	●	PFL + XAI	92%	91%	90.5%

**Table 3:** Challenges addressed by our proposed PFL-XAI model in EEG-based depression detection.

Challenge	Previous Solutions	Limitations	Our Proposed Solution
Data Privacy	Centralized models [37]	Data centralization poses privacy risks	PFL ensures data remains local
Explainability	Black-box models [38]	Limited interpretability for clinicians	Full integration of XAI for better feature explainability
Personalization	Generalized models [39]	Lack of patient-specific tuning	Personalized learning for each patient through PFL
Scalability	High-performance models [40]	High computational costs	Optimized communication for scalable PFL
Heterogeneous EEG Data	CNN + LSTM [41]	Low accuracy with heterogeneous datasets	Adaptation to multiple data sources through PFL
Real-Time Application	CNN + LSTM [42], Reinforcement Learning [43]	High computational costs limit real-time use	Efficient real-time processing with personalized models

**Table 4:** Comparative analysis of federated learning (FL), centralized learning (CL), and proposed PFL-XAI approach in EEG-based depression detection.

Characteristic	Centralized Learning (CL)	Federated Learning (FL)	Our Proposed PFL-XAI Approach
Data Handling	Centralized; data stored on a remote server	Decentralized; data remains local	Decentralized with personalization
Privacy	Low (data uploaded to a central server)	High (data remain on local devices)	High (enhanced privacy with PFL)
Personalization	Low (single model for all patients)	Moderate (different models for groups)	High (personalized per patient)
Explainability	Low	Low	High (explainable AI integration)
Scalability	Moderate	High	High
Performance Overhead	Moderate	High (frequent communication required)	Moderate (optimized communication)
Model Accuracy	85–90%	82–88%	92% (higher due to PFL)

**Table 5:** Conceptual impact of personalized federated learning (PFL) on EEG-based depression detection compared to standard AI approaches. This avoids direct cross-dataset comparisons and emphasizes methodological benefits.

Aspect	Standard AI Models	With PFL Integration	Clinical/Practical Benefit
EEG-Based Depression Detection	Accuracy varies across datasets; limited privacy protection	Improved personalization; privacy preserved during training	Reliable and secure depression detection in diverse populations
Mental Health Monitoring	Generalized predictions not tailored to individuals	Patient-specific adaptation; supports longitudinal data	More accurate monitoring and relapse prediction
Explainability	Black-box predictions with limited interpretability	Integration with explainable AI (XAI) frameworks	Clinician trust and decision support enhanced
Scalability	High communication and computational costs in federated settings	Optimized communication strategies in PFL	Feasible for multi-center mental health studies
Data Heterogeneity	Poor generalization across different EEG sources	Robust handling of diverse acquisition setups	Consistent predictions across devices and institutions

**Table 6:** Performance metrics of EEG-based depression detection models.

Model	Accuracy (%)	Precision (%)	Recall (%)	F1-Score (%)	Computational Cost	Explainability	Personalization
CNN + LSTM	88%	85%	87%	86%	High	Low	None
Federated Learning (FL)	85%	83%	84%	83%	High	Low	Moderate
Reinforcement Learning	75%	72%	76%	74%	Moderate	Low	None
Explainable AI (XAI)	82%	81%	80%	81%	High	Moderate	Low
Meta-Learning	86%	85%	86%	85%	High	Moderate	High
Federated Learning with XAI	87%	85%	88%	87%	Moderate	Moderate	High
Our Proposed PFL-XAI Model	92%	91%	93%	90.5%	Moderate	High	High

**Table 7:** Statistical validation of CNN-LSTM model for depression relapse prediction. Reported values are mean ± 95% confidence intervals (CIs) with associated *p*-values.

Metric	Value (95% CI)	*p*-Value
Accuracy	89.2% (87.6–90.8)	<0.01
Precision	88.1% (86.5–89.7)	<0.01
Recall	87.5% (85.9–89.1)	<0.01
F1-Score	87.8% (86.2–89.4)	<0.01

## Data Availability

The dataset is available at the following link: https://fcon_1000.projects.nitrc.org/indi/cmi_healthy_brain_network/Data.html (Healthy Brain Network Dataset), which was accessed on 20 August 2025.

## Abbreviations

EEG	Electroencephalography
HBN	Healthy Brain Network
PFL	Personalized Federated Learning
FL	Federated Learning
XAI	Explainable Artificial Intelligence
LRP	Layer-Wise Relevance Propagation
AWEF	Adaptive Wavelet Entropy Filtering
CNN	Convolutional Neural Network
LSTM	Long Short-Term Memory
ViT	Vision Transformer
BCE	Binary Cross-Entropy
AUC	Area Under the Curve
MAE	Mean Absolute Error
RMSE	Root Mean Squared Error
MAPE	Mean Absolute Percentage Error
MCC	Matthews Correlation Coefficient
ICA	Independent Component Analysis
EMD	Empirical Mode Decomposition
CCA	Canonical Correlation Analysis
MFQ	Mood and Feelings Questionnaire
CBCL	Child Behavior Checklist
DSM-5	Diagnostic and Statistical Manual of Mental Disorders, 5th Edition
TP/TN/FP/FN	True Positive, True Negative, False Positive, False Negative
ROC	Receiver Operating Characteristic
NPV	Negative Predictive Value
